# Two-eyed seeing as a philosophy to facilitate communication between traditional indigenous cultural practitioners with psychiatry and other mental health practitioners

**DOI:** 10.1192/j.eurpsy.2021.1818

**Published:** 2021-08-13

**Authors:** L. Mehl-Madrona, B. Mainguy

**Affiliations:** 1 Medical Arts And Humanities Program, University of Maine, Orono, United States of America; 2 Education Division, Coyote Institute - Canada, Ottawa, Canada

**Keywords:** Indigenous people, two-eyed seeing, mental health, philosophy

## Abstract

**Introduction:**

A communication gap exists between psychiatry and indigenous people about views of mind and mental health, which often becomes an obstacle to optimal care and a source of distrust.

**Objectives:**

We aimed to explore the utility of the concept of two-eyed seeing for facilitating communication among traditional cultural practitioners (TCP) and conventional mental health practitioners (CMHP).

**Methods:**

“Two-eyed seeing” is spreading across North America as a metaphor for explanatory pluralism. Albert Marshall, a M’iqmaq from Nova, Scotia, Canada, developed this traditional concept (eptuamptamuk in M’iqmaq) to speak to the idea that indigenous knowledge is as valid as contemporary science for conceptualizing phenomena. We taught the concept to 100 practitioners, equally balanced between CMHP’s and TCP’s, and obtained ongoing feedback about the results of their applying these ideas to their ongoing collaborations. Qualitative research methods were used to evaluate this feedback.

**Results:**

Using the two-eyed seeing concept allowed CMHP’s to better listen to TCP’s descriptions of their concepts of mind and of mental suffering. TCP’s felt more respected by CMHP’s. While concepts such as spirit visitation, the breaking of taboos, and intergenerational curses are inherently foreign to CMHP’s, the two-eyed seeing concept allowed them to bracket these ideas as interesting and to interact with the TCP in a more productive way, while allowing them to observe the effects of the TCP’s interventions in a less judgmental way.

**Conclusions:**

Two-eyed seeing allowed a rich dialogue between CMHP’s and TCP’s that enabled each to appreciate the other’s perspectives, leading to greater cooperation and collaborative treatment. Outcomes improved.

**Disclosure:**

No significant relationships.

